# Postmenopausal osteoporosis: Effect of moderate-intensity treadmill exercise on bone proteomics in ovariectomized rats

**DOI:** 10.3389/fsurg.2022.1000464

**Published:** 2023-01-06

**Authors:** Yong-Jie Yang, Ye Li, Li Gao

**Affiliations:** College of Sports and Health, Shandong Sport University, Ji’nan, China

**Keywords:** ovariectomized rats, moderate-intensity treadmill exercise, proteomics, postmenopausal osteoporosis, bone proteomics

## Abstract

**Objectives:**

This study aimed to identify the key proteins in the bone mass of ovariectomized (OVX) rats after a period of regular moderate-intensity treadmill exercise and to investigate their effects using tag mass spectrometry and quantitative proteomics with a view to improving the understanding and treatment of postmenopausal osteoporosis.

**Methods:**

Sixty three-month-old female Sprague-Dawley tats of specific-pathogen-free grade were randomly and equally divided into a sham operation group, ovariectomized group (OVX) and ovariectomized combined exercise (OVX + EX) group, and the latter took moderate-intensity treadmill exercise for 17 weeks. After this period of time, body composition and bone density were measured using dual-energy x-ray absorptiometry, and serum bone metabolism indicators were measured using an enzyme immunoassay. In addition, the bone microstructure was examined using micro-computed tomography and scanning of the femur, and femur proteins were subject to proteomic analysis.

**Results:**

Compared with the rats in the OVX group, the bone metabolism indicators in the OVX + EX group decreased significantly, femur bone density increased significantly, the number of the trabeculae increased, and continuity was higher. In the OVX + EX group, 17 proteins were significantly upregulated and 33 significantly downregulated. The main gene ontology and signaling pathways enriched by the proteins were identified as the tumor necrosis factor-mediated signaling pathways. The protein-protein interaction network identified the key proteins, and the correlation analysis of these proteins and the bone parameters found histone deacetylase 8(HDAC8) and leucine-rich transmembrane and O-methyltransferase domain containing (LRTOMT) and trimethylguanosine synthase 1(TGS1) and ankyrin repeat domain 46(ANKRD46) to be the key targets of exercise in relation to postmenopausal osteoporosis.

**Conclusion:**

Moderate-intensity treadmill exercise significantly improved the bone mass of OVX rats, and differentially expressed proteins, such as HDAC8 and LRTOMT and TGS1 and ANKRD46, could be the target of moderate-intensity treadmill exercise.

## 1. Introduction

Postmenopausal osteoporosis (PMOP) is a common and frequently occurring disease among postmenopausal women. It is a systemic bone disease, characterized by decreased bone mass, disrupted bone microarchitecture, and increased bone fragility, where bones are prone to fracture. Human and animal experiments have demonstrated that early exercise intervention can play a role in preventing PMOP and promoting the recovery of bone mass. A previous study found that moderate-intensity treadmill exercise improved the bone mineral density (BMD) and bone microstructure of ovariectomized (OVX) rats, but its mechanism needs further investigation (1). Proteomics is an important tool for identifying new human disease biomarkers and new targets for drug treatment by analyzing the changes in proteins in target tissues. In recent years, many proteomic studies have been conducted on bone-related cells or tissues, such as mononuclear cells, plasma, and serum, to explore the pathogenesis and monitoring indicators of PMOP (2–5). In 2017, the first comprehensive human monocyte proteome knowledge base was developed (2). Bone tissue is the main pathogenesis site of PMOP, and the occurrence of the disease is often accompanied by changes in protein expression. Therefore, further study of the pathogenesis of PMOP through an in-depth investigation of the expression of the bone tissue protein group is required. In particular, the protein changes in bone tissue produced during sports activities are undetermined. In this study, tandem mass tag (TMT) mass spectrometry and quantitative proteomics were used to screen the differentially expressed proteins in the femurs of OVX osteoporotic rats after a moderate-intensity treadmill exercise intervention. The aim was to explore the preventative effects of exercising on PMOP and its molecular mechanism. It is hoped that this will provide a scientific basis for the clinical exploration of new prevention methods and rehabilitation treatment for patients with PMOP.

## 2. Animals and methods

### 2.1. Animals and groupings

Sixty specific-pathogen-free three-month-old female Sprague Dawley rats, weighing 272 ± 14 g, were purchased from Beijing Vitong Lihua Laboratory Animal Technology Co. Ltd. (License No.: SCXK (J) 2016-0006). They had been raised in a single cage at an environmental temperature of 22°C–26°C and a relative humidity of 45%–70%, with free access to food in a 12-h light/12-h dark environment and fed a standard rodent diet. The rats were randomly divided into the OVX (*n* = 40) and the sham operation (SHAM; *n* = 20) groups. Bilateral dorsal ovariectomies were performed in the OVX group. One week after surgery, 20 rats from the OVX group were randomly selected and placed into the OVX combined exercise (OVX + EX) group. The body weight of the rats was weighed weekly, and the food intake of the rats was recorded daily.

### 2.2. Model preparation

All the rats were fasted for 10–12 h. All the experimental rats were anesthetized using an intraperitoneal injection of a general anesthetic agent [3% pentobarbital sodium (1 ml/kg)], laterally positioned, and disinfected with iodine and alcohol. A longitudinal incision was made 0.5–1.0 cm adjacent to the midline of the bilateral back of the rats. The skin, fascia, and muscle were separated to both sides, and the weakest parts of the left and right abdominal muscles were opened. The pink ovaries were visible below the kidneys on both sides, and the ovaries were gently lifted. After ligation, the ovaries were removed, and the skin was sutured. In the SHAM group, a sham operation was performed in which the hair was removed, an incision was made on the skin in the center of the back in the same manner as in the OVX group, and the site was sutured after removing the large, white adipose tissue adjacent to the ovaries.

### 2.3. Exercise program

The rats were rested for 1 week postoperatively, iodine tincture was smeared on the wound daily, and penicillin injections were administered for three consecutive days to prevent infection. In the second week after the operation, the rats in OVX + EX group received adaptive treadmill training (ZH-PT Animal Experiment Treadmill, Huaibei Zhenghua Biological Instrument Equipment, Anhui Province, China) at a fixed time every day, and in the third week after the operation, the rats were trained at 20 m/min for 60 min until the end of moderate intensity treadmill exercise program. All training occurred in the morning, 5 days per week for 17 weeks.

### 2.4. Detection of body composition and bone metabolism, mineral density, and microstructure

The OVX + EX rats were sampled 24–36 h after their final training session. The rats from each group were weighed and anesthetized before the sampling. A Lunar Prodigy dual-energy x-ray bone densitometer (General Electric, Boston, MA, USA) was used to scan and calculate the percentage of muscle and fat in the whole body. The abdominal cavity was then quickly opened to expose the abdominal aorta, take blood, separate the serum, and measure the contents of serum tartrate-resistant phosphatase 5b (TRACP 5b) and osteocalcin (OCN) using an enzyme immunoassay (purchased from IDS Immunodiagnostic Systems Limited, London, UK).

Subsequently, the rats were euthanized; the left femur were separated; and the soft tissues were removed. The tissues were wrapped in gauze soaked with normal saline and placed in a test tube. After labeling, the tissues were frozen at −80°C for the determination of the BMD. The Norland XR-600 bone densitometer (NORLAND CooperSurgical Company, Connecticut, USA) was used to measure the BMD and bone mineral content in the distal femur. The right femur was separated; Four right femurs were randomly selected from each group then fixed with 4% paraformaldehyde to observe the bone microstructure. The distal femurs were scanned using the SkyScan 1172 Microtomography (m-CT, SkyScan 1172, Bruker, Belgium) with a voltage of 80 kV, a current of 112 µA, and a pixel density of 9.00 × 9.00 µm. After scanning, the samples were reconstructed. The region of interest was located between the disappearance of the distal growth plate of the femur and 100 layers above it. The image information was extracted and the image binarization was completed by the computer. Finally, a quantitative analysis was carried out using software to obtain the indicators for the microstructure of the distal femurs of the rats including Bone volume fraction (BV/TV)%, Mean number of objects per slice, Closed porosity (percent)% and many more. At the same time, three-dimensional reconstruction images were created. The rest of the right femur was frozen in liquid nitrogen and stored at −70°C.

### 2.5. Determination and analysis of the bone tissue protein groups

#### 2.5.1. Sample preparation

In each group, we randomly mixed five samples into one sample for the TMT proteome experiment. That is, three mixed bone samples in each group were prepared for further proteome analysis. Cancellous bone of the same mass at the same site of the right distal femur (3 mm to the rear and above where the growth plate disappears) from five rats was mixed into one sample (0.5 g) and ground with liquid nitrogen. 5 times volume of TCA/acetone (1:9) was added to the powder and mixed by vortex. The mixture was placed at −20°C for 4 h, and centrifuged at 6000 g for 40 min at 4°C. The supernatant was discarded. The pre-cooling acetone was added and washed for three times. The precipitation was air dried. 30 times volume of SDT buffer was added to 20–30 mg powder, mixed and boiled for 5 min. The lysate was sonicated and then boiled for 15 min. After centrifuged at 1,4000 g for 40 min, the supernatant was filtered with 0.22 µm filters. The filtrate was quantified with the BCA Protein Assay Kit (Bio-Rad Laboratories, Hercules, CA, USA). The sample was stored at −80°C.

#### 2.5.2. Filter-aided sample preparation (FASP digestion)

200 µg of proteins for each sample were incorporated into 30 µl SDT buffer (4% SDS, 100 mM DTT, 150 mM Tris-HCl pH 8.0). The detergent, DTT and other low-molecular-weight components were removed using UA buffer (8 M Urea, 150 mM Tris-HCl pH 8.0) by repeated ultrafiltration (Microcon units, 10 kD). Then 100 µl iodoacetamide (100 mM IAA in UA buffer) was added to block reduced cysteine residues and the samples were incubated for 30 min in darkness. The filters were washed with 100 µl UA buffer three times and then 100 µl 100 mM TEAB buffer twice. Finally, the protein suspensions were digested with 4 µg trypsin (Promega) in 40 µl TEAB buffer overnight at 37°C, and the resulting peptides were collected as a filtrate. The peptide content was estimated by UV light spectral density at 280 nm.

#### 2.5.3. TMT labeling

100 µg peptide mixture of each sample was labeled using TMT reagent according to the manufacturer's instructions (Thermo Fisher Scientific, Waltham, MA, USA).

#### 2.5.4. Peptide fractionation with high pH reversed-phase

According to the manufacturer's instructions, increasing the acetonitrile step-gradient elution, the TMT-labeled digest samples into 10 fractions fractionated with a Pierce high pH reversed-phase fractionation kit (Thermo Fisher Scientific, Waltham, MA, USA).

#### 2.5.5. Mass spectrometry

##### 2.5.5.1. HPLC

Each fraction was injected for nanoLC-MS/MS analysis. The peptide mixture was loaded onto a reverse phase trap column (Thermo Scientific Acclaim PepMap100, 100 µm*2 cm, nanoViper C18) connected to the C18-reversed phase analytical column (Thermo Scientific Easy Column, 10 cm long, 75 µm inner diameter, 3 µm resin) in buffer A (0.1% Formic acid) and separated with a linear gradient of buffer B (84% acetonitrile and 0.1% Formic acid) at a flow rate of 300 nl/min controlled by IntelliFlow technology.

##### 2.5.5.2 LC-MS/MS analysis

The LC-MS/MS analysis was completed with a Q Exactive mass spectrometer (Thermo Fisher Scientific, Waltham, MA, USA) and it was coupled to Easy nLC for 60 min. The mass spectrometer was performed in positive ion mode. Using a data-dependent top10 method can obtain the MS data, and the most abundant precursor ions were selected from the scan of 300–1,800 m/z. An automatic gain control (AGC) target was 3e6, and the maximum inject time was 10 ms. Dynamic exclusion duration was 40 s. Survey scans were obtained with the resolution of 70,000 at m/z 200 and the resolution for the HCD spectra was 17,500 with m/z 200, 35,000 at m/z 200, and the width of isolation was 2 m/z. The normalized collision energy was 30 and the under fill ratio specified the minimum percentage of the target value was defined as 0.1%. The instrument was performed by using peptide recognition mode enabled. The LC–MS/MS spectra were searched by using a MASCOT engine which was embedded into Proteome Discoverer 1.4. The original mass spectrometry data was a RAW file, and Mascot 2.2 and Proteome Discoverer 1.4 software were used for the database search, identification, and quantitative analysis. The MS proteomics data have been deposited to the ProteomeXchange Consortium *via* the PRIDE partner repository with the dataset identifier PXD034812.

### 2.6. Bioinformatics analysis

The differentially expressed proteins were screened according to the criteria of a more than 1.2-fold change in expression (upregulation more than 1.2-fold or downregulation less than 0.83-fold) and a *P* value <0.05. Blast2Go (https://www.blast2go.com) was used for the gene ontology (GO) annotation of the differential proteins, and Kyoto Encyclopedia of Genes and Genomes (KEGG) Automatic Annotation Server software was used for the KEGG pathway annotation. Fisher's exact test was used to compare the distribution of each GO classification or KEGG pathway in the differential protein set and overall protein set, and an enrichment analysis of the GO annotation or KEGG pathway annotation was conducted on the differential protein set. The protein interaction network was constructed using the STRING 11.5 database (https://cn.string-db.org/) for screened differential proteins to further identify key proteins in the network.

### 2.7. Data analysis

Statistical analyses were performed using SPSS 23.0. All data results were expressed as mean ± standard deviation (X ± SD). BMD of distal femur were analyzed by analysis of covariance(ANCOVA). Other indicators were compared using one-way analysis of variance, and the correlation between bone microstructure parameters and the expression of key differential proteins was analyzed using Spearman's correlation analysis, factor loading (rotated) matrix analysis and generalized linear regression analysis with *P* < 0.05 denoting a significant difference and *P* < 0.01 a very significant difference.

## 3. Study results

### 3.1. Effects of moderate-intensity treadmill exercise on body composition and bone mineral density, microstructure, and metabolism indicators in ovariectomized rats

**Figure 1:**
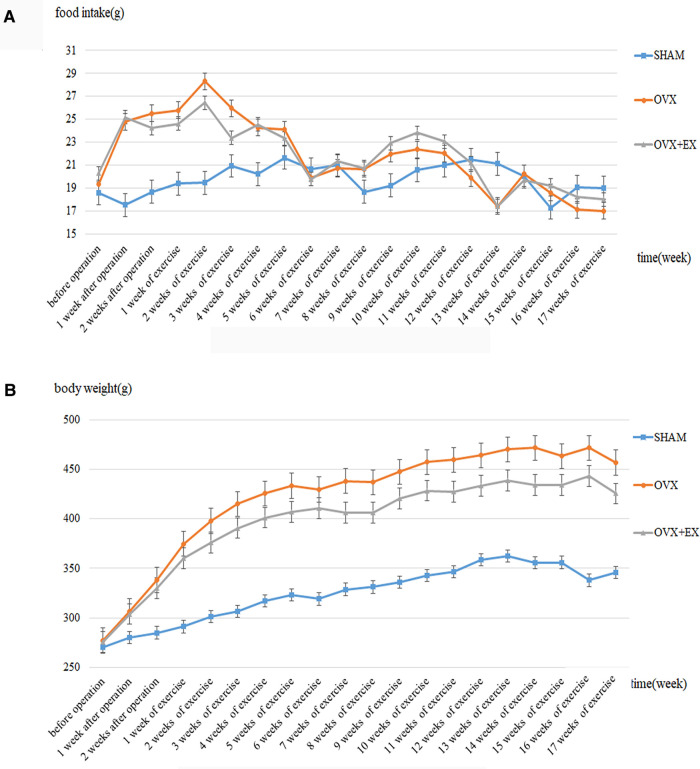
Dynamic change of the food intake and the body weight in rats. **(A)** Dynamic change of the food intake in rats; **(B)** Dynamic change of the body weight in rats.

Ovariectomy caused an increase in diet and weight of rats (Figure 1), Compared with the sham group, the weight and fat content of the OVX rats increased significantly, whereas the muscle content and BMD decreased significantly. Compared with the OVX group, the weight and fat content of the OVX + EX rats decreased significantly, whereas the muscle content and BMD were significantly higher than those in the OVX group (Table 1). The results of covariance analysis showed that body weight had a significant impact on bone mineral density, but excluding the impact of body weight, it was found that exercise intervention significantly increased the bone mineral density of rats. This indicated that the ovariectomy led to an increase in body weight and body fat as well as bone loss, whereas exercise helped to slow down the decline in muscle content and bone density, partially preventing the bone loss caused by the ovariectomy.

**Table 1:** Comparison of body weight, body composition, bone mineral density and bone metabolism indicators of rats in each group.

Indicators	SHAM	OVX	OVX + EX
body weight (g)	345.89 ± 34.05	456.75 ± 47.55**	425.43 ± 35.12**^ΔΔ^
Fat content of the whole body (%)	54.18 ± 6.86	65.37 ± 8.79**	58.51 ± 6.18^ΔΔ^
Muscle content of the whole body (%)	41.42 ± 7.04	29.40 ± 6.84**	38.36 ± 6.38^ΔΔ^
Distal femur BMD (g/cm^2^)	0.28 ± 0.03	0.23 ± 0.02**	0.25 ± 0.02**^ΔΔ^
TRACP 5b (U/L)	1.45 ± 0.12	1.72 ± 0.43*	1.45 ± 0.09^Δ^
OCN (ng/ml)	369.86 ± 103.36	572.25 ± 136.82*	385.89 ± 124.43^Δ^

Compared with SHAM, * means significant *P* < 0.05, ** means significant *P* < 0.01; Compared with OVX, ^Δ^ indicates significant *P* < 0.05, ^ΔΔ^ indicates significant *P* < 0.01.

**Figure 2:**
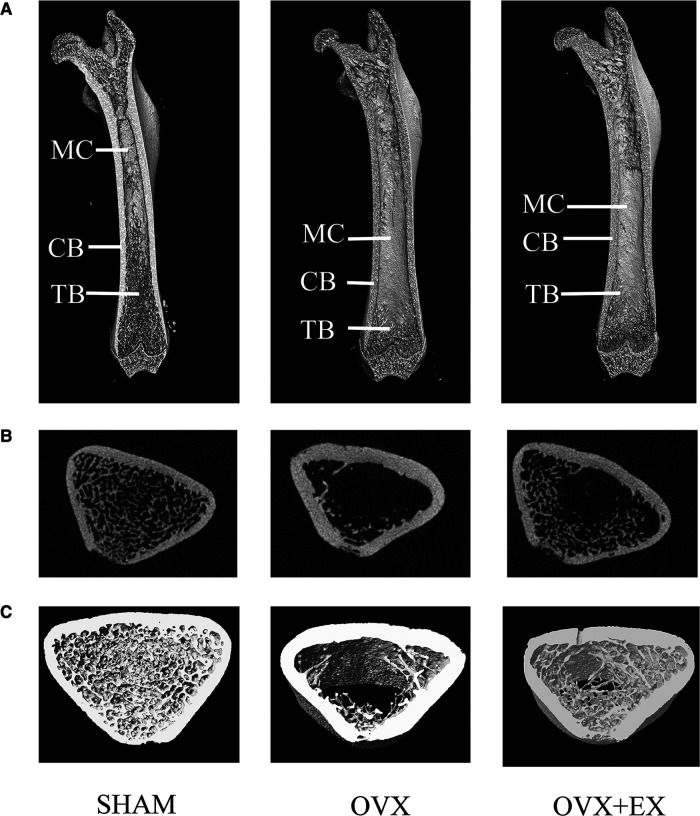
Two-dimensional structure of the longitudinal and transverse sections of the femur and the three-dimensional (3D) reconstruction of cancellous bone of the distal femur in the region of interest: (**A**) the longitudinal section of the femur; (**B**) the transverse section of the distal femur; and (**C**) 3D reconstruction of bone. (Representative micro-computed tomography pictures are shown here). MC, Marrow Cavity; CB, cortical bone; TB, trabecular bone.

The results of the two-dimensional and 3D structural reconstruction of the femur revealed that, compared with the sham group, the femoral trabecula microstructures in the OVX rats were severely damaged, with few trabeculae and large spaces and increased bone marrow cavity volume between them. There was an increased number of trabeculae and higher continuity and smaller intertrabecular space, lengthen cancellous bone and shorten bone marrow cavity in the OVX + EX rats compared with the OVX group (Figure 2). The results demonstrated that the volume of cancellous bone in the OVX rats was reduced, and the number and thickness of trabeculae decreased. However, moderate-intensity exercise increased the volume of cancellous bone and the number of trabeculae, reduced the separation degree of bone trabeculae, and improved the bone microstructure of femoral cancellous bone.

Compared with the sham group, the serum TRACP 5b and OCN in the OVX group were significantly higher than those in the sham group. Compared with the OVX group, TRACP 5b and OCN were significantly lower in the OVX + EX group (Table 1). The results indicated that the ovariectomy promoted a high turnover rate and a strengthen bone resorption in bone metabolism, while moderate-intensity treadmill exercise lowered the turnover rate and bone resorption.

### 3.2. Effect of moderate-intensity treadmill exercise on the femoral proteomics of ovariectomized rats

**Figure 3:**
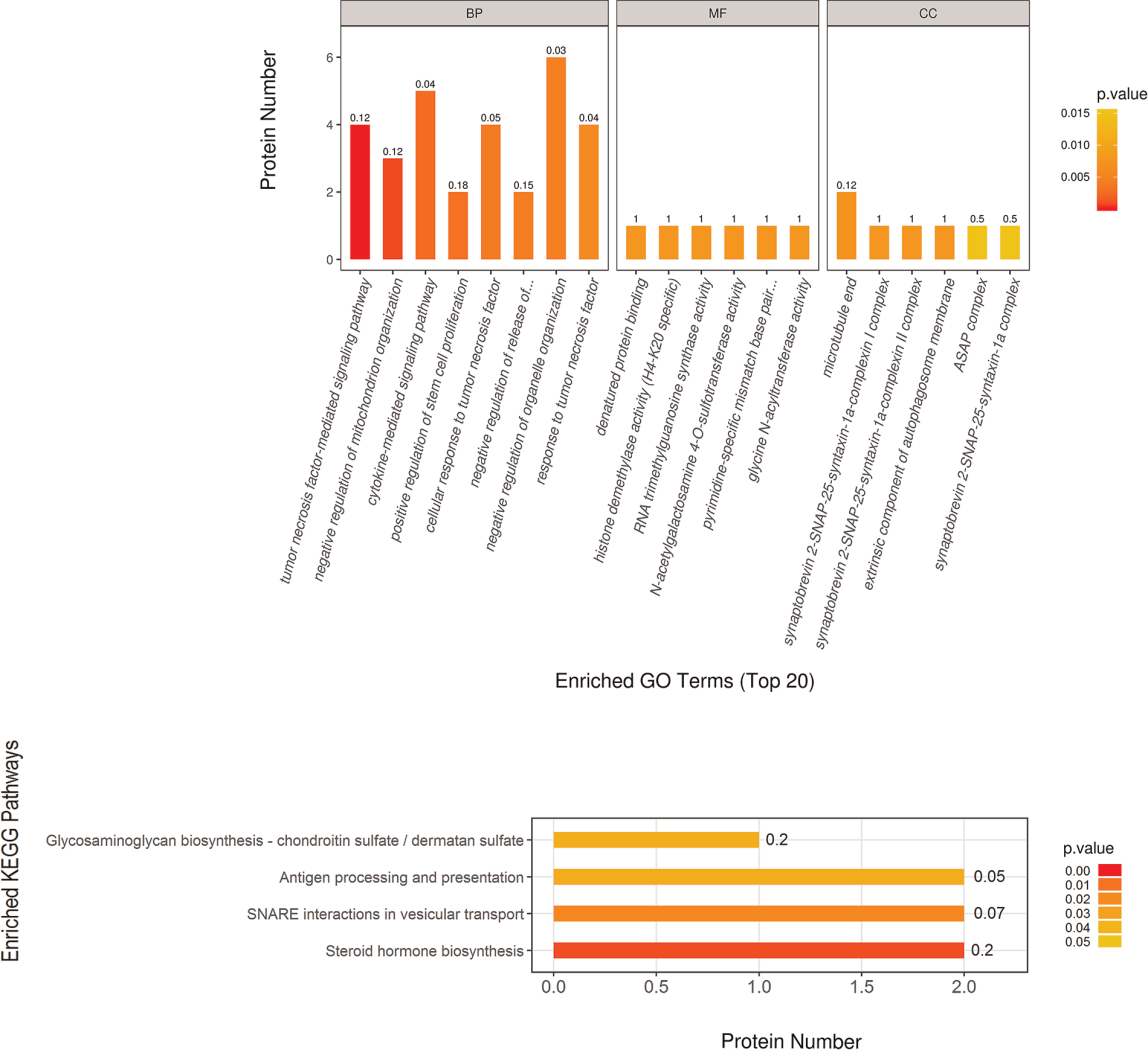
Significantly enriched GO terms and KEGG pathways, showing higher protein translating levels in the OVX + EX group than the OVX group.

**Figure 4:**
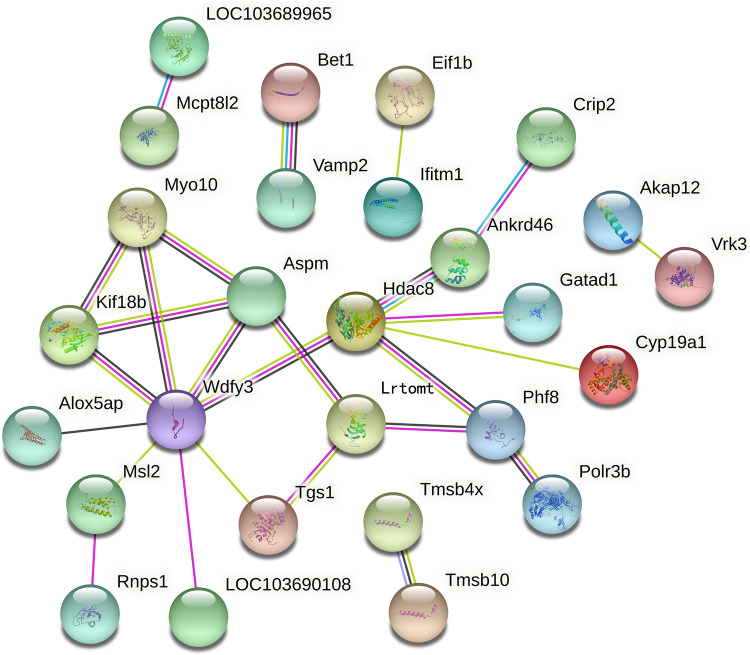
Key proteins in the protein–protein interaction network in the OVX + EX and OVX groups.

Using the TMT quantitative proteomics technique, a total of 6,496 proteins identified separately in either group of ovariectomized rats. Compared with the OVX group, the OVX + EX group had 50 differential proteins, including 17 upregulated proteins and 33 downregulated proteins. The enrichment analysis of the 50 differential proteins revealed that the main GO and signaling pathways enriched by these proteins were the tumor necrosis factor (TNF)-*α*-mediated signaling pathways (Figure 3). In the protein–protein interaction (PPI) network constructed by the differential proteins, the more connections these proteins had with other proteins, the greater the interaction ability. Through the PPI network, it was determined that ten proteins [WD repeat and FYVE domain containing 3 (WDFY3), histone deacetylase 8(HDAC8), abnormal spindle microtubule assembly (ASPM), kinesin family member 18B (KIF18B), leucine rich transmembrane and O-methyltransferase domain containing (LRTOMT), myosin X (MYO10), PHD finger protein 8 (PHF8), ankyrin repeat domain 46 (ANKRD46), MSL complex subunit 2 (MSL2), and trimethylguanosine synthase 1 (TGS1)] had an interaction relationship of ≥4 with other proteins (Figure 4).

The correlation analysis between these ten proteins and the bone microstructure parameters demonstrated that HDAC8, LRTOMT, TGS1 and ANKRD46 were significantly correlated with multiple parameters, including bone microstructure and bone density (Table 2). After factor load matrix analysis, these ten proteins were divided into two groups (Table 3), namely, factor 1 (F1) and factor 2 (F2). The generalized linear regression analysis of the two factors with bone microstructure parameters and distal femoral BMD showed that F2 had significant correlation. Although F1 displayed significant correlation in Tb.Sp(pl) and Tb.Sp(rd), both of *β*, the standardized coefficients, showed *β*F1 < *β* F2 (Table 4), Which implied that F2, namely HDAC8, LRTOMT, TGS1 and ANKRD46, is more closely related to bone. This result is consistent with spearman correlation analysis. Compared with the OVX rats, proteomics revealed that the expressions of HDAC8 and LRTOMT and TGS1 were significantly decreased, the expressions of ANKRD46 were significantly increased in the femurs of the OVX + EX rats (Table 5). The results indicated that HDAC8, LRTOMT, TGS1 and ANKRD46 are important candidate biomarkers for the effect of exercise on PMOP.

**Table 2:** Correlation analysis between Top 10 differential proteins of protein-protein interaction network and bone microstructure parameters.


	PHF8	HDAC8	ANKRD46	ASPM	KIF18B	MSL2	MYO10	TGS1	WDFY3	LRTOMT
Bone volume fraction (BV/TV)%	−.473	−.917**	.591*	−.177	−.266	−.296	−.296	−.650*	−.296	−.866*
Mean number of objects per slice	−.473	−.917**	.591*	−.177	−.266	−.296	−.296	−.650*	−.296	−.866*
Trabecular thickness [Tb.Th (pl)] µm	−.473	−.917**	.591*	−.177	−.266	−.296	−.296	−.650*	−.296	−.866*
Trabecular separation [Tb.Sp (pl)] µm	.473	.917**	−.591*	.177	.266	.296	.296	.650*	.296	.866*
Trabecular separation [Tb.Sp (rd)] µm	.473	.917**	−.591*	.177	.266	.296	.296	.650*	.296	.866*
Trabecular number [Tb.N (pl)] µm^−1^	−.473	−.917**	.591*	−.177	−.266	−.296	−.296	−.650*	−.296	−.866*
Trabecular number [Tb.N (rd)] µm^−1^	−.473	−.917**	.591*	−.177	−.266	−.296	−.296	−.650*	−.296	−.866*
Diameter of trabecular bone [Tb.Dm (rd)] µm	−.473	−.917**	.591*	−.177	−.266	−.296	−.296	−.650*	−.296	−.866*
Average of Trabecular Bone Pattern factor (Tb.Pf) µm^−1^	.473	.917**	−.591*	.177	.266	.296	.296	.650*	.296	.866*
Mean total cross-sectional tissue perimeter µm	−.473	−.917**	.591*	−.177	−.266	−.296	−.296	−.650*	−.296	−.866*
Distal femur BMD (g/cm^2^)	−.473	−.917**	.591*	−.177	−.266	−.296	−.296	−.650*	−.296	−.866*

*Means significant *P* < 0.05, **means significant *P* < 0.01.

**Table 3:** Factor loading (rotated) matrix of key proteins.^a^


	Factor1 (F1)	Factor2 (F2)
PHF8	0.652	
HDAC8		0.932
ANKRD46		−0.75
ASPM	0.927	
KIF18bB	0.894	
MSL2	0.909	
MYO10	0.922	
TGS1		0.779
WDFY3	0.87	
LRTOMT		0.928

Extraction method: principal component analysis; Rotation method: varimax with Kaiser normalization.

^a^ Means Maximum Iterations for convergence is 3.

**Table 4:** Generalized linear regression between key proteins and parameters of distal femur.


	*p*F1	*β*F1	*p*F2	*β*F2
Distal femur BMD (g/cm^2^)	0.4120	0.1120	0.0001	−0.9140
Bone volume fraction (BV/TV)%	0.1445	0.2340	0.0002	−0.8674
Mean number of objects per slice	0.1320	−0.1524	0.0000	−0.9490
Trabecular thickness [Tb.Th (pl)] µm	0.4242	−0.0851	0.0000	−0.9485
Trabecular separation [Tb.Sp (pl)] µm	0.0010	0.3321	0.0000	0.9199
Trabecular separation [Tb.Sp (rd)] µm	0.0443	0.1995	0.0000	0.9458
Trabecular number [Tb.N (pl)] µm^−1^	0.1768	0.2100	0.0002	−0.8781
Trabecular number [Tb.N (rd)] µm^−1^	0.5586	0.0760	0.0000	−0.9240
Diameter of trabecular bone [Tb.Dm (rd)] µm	0.4240	−0.0851	0.0000	−0.9485
Average of Trabecular Bone Pattern factor (Tb.Pf) µm^−1^	0.5980	0.0577	0.0000	0.9467
Mean total cross-sectional tissue perimeter µm	0.3714	0.1239	0.0001	−0.9103

*p*F1 and *p*F2: *P*-value of regression factor score 1 and 2 for analysis 1, *P* < 0.05 means significant: *β*F1and *β*F2: Standardized Coefficients of regression factor score 1 and 2 for analysis 1.

**Table 5:** Expression information of key proteins HDAC8, LRTOMT, TGS1 and ANKRD46.


Name of proteins	Protein symbols	Fold change (log_2_FC) OVX + EX vs. OVX
histone deacetylase 8	HDAC8	0.800507↓^Δ^
leucine rich transmembrane and O-methyltransferase domain containing	LRTOMT	0.716366↓^Δ^
trimethylguanosine synthase 1	TGS1	0.721547↓^Δ^
ankyrin repeat domain 46	ANKRD46	1.207508↑^Δ^

Compared with OVX, ^Δ^ indicates significant *P* < 0.05, ↓ indicates down-regulation, ↑ indicates up-regulation.

## 4. Discussion

### 4.1. Effect of moderate-intensity treadmill exercise on the bone mass of ovariectomized rats

Postmenopausal osteoporosis is a disease characterized by low bone mass and the gradual destruction of the bone microstructure, which leads to an increased risk of fracture. The OVX rat is a typical experimental model used to study PMOP caused by female estrogen deficiency. In this study, the PMOP rat model was replicated using the classic ovariectomy method (6). Compared with the sham group, the OVX rats exhibited increased body weight and fat content and decreased femoral BMD, indicating the successful modeling of OVX osteoporosis rats. A large number of studies have found that exercise has an early preventive effect on PMOP (7–10). In this experiment, we observed that after 17 weeks of moderate-intensity treadmill exercise, body weight and body fat decreased, muscle content increased, BMD and cancellous bone structure of distal femur improved, and the number of bone trabeculae increased in ovariectomized rats. The findings of these studies are consistent with the results of the current study, that is, physical exercise has positive preventive and therapeutic effects on PMOP.

Serum bone metabolism indicators reflect the state of bone formation, absorption, and remodeling. The average OCN and TRACP 5b levels in postmenopausal women with osteoporosis was significantly higher than that of women without osteoporosis in human experiment (11). And there was a negative correlation between high bone turnover rate and BMD. Several studies have reported that not only did the OVX osteoporosis rats present with osteoporosis but the biochemical markers of their bone turnover also exhibited a high turnover rate (12–15), with biasing to bone loss in bone remodeling (16). The results of the present study showed that both the TRACP 5b and OCN of the OVX group rats were significantly increased, indicating that estrogen deficiency resulted in high bone turnover, destroying the structure of the bone trabeculae and reducing their number and thickness, thereby reducing bone strength and bone density and, eventually, leading to the occurrence of osteoporosis.

Reports showed there was lower the number of TRACP 5b-positive osteoclasts and osteoclast differentiation in the tibia of OVX mice trained by treadmill exercise (17). Exercise reduced the level of bone turnover markers and the expression of interleukin (IL)-1, IL-6, and cyclooxygenase-2 of the bone marrow in OVX rats, and increased the bone mass of OVX rats by inhibiting bone trabecular bone resorption and increasing bone formation besides (18, 19). This study demonstrated that moderate-intensity treadmill exercise reduced high bone turnover in OVX rats, indicating that exercise could improve bone mass in OVX rats by inhibiting bone resorption and increasing bone formation. However, the mechanism underlying the improvements in bone metabolism and bone mass as a result of physical exercise and the pathways and factors involved requires further study.

### 4.2. Effect of moderate-intensity treadmill exercise on the femoral proteomics of ovariectomized rats

Postmenopausal osteoporosis is a systemic disease with a complex pathogenesis that affects bone metabolism. In recent years, proteomics has been increasingly applied to the study of bone physiology and pathology, especially PMOP (20–22). The main protein markers of osteoporosis gained from peripheral blood mononuclear cells (23, 24), cytoplasm (25, 26), or serum (27–29) can't fit well with those obtaining from human bone tissue samples (5), suggesting that blood proteomics might not truly reflect the molecular mechanism of osteoporosis.

Several studies have demonstrated that exercise affects bone turnover and regulates bone formation through a variety of signaling pathways, such as receptor activator of nuclear factor kappa-*Β* ligand (RANKL)/RANK/osteoprotegerin pathway (30–32). This study revealed that the TNF-α-mediated signaling pathway plays a key role in the improvement of bone mass through exercise in OVX rats. Consistent with previous reports, a weight-loss program combined with aerobic exercise reduces adipose tissue, which may lead to reduced TNF-α activity and a subsequent reduction in bone loss (33). According to osteoimmunology studies, the bone and immune systems are closely related through many shared regulatory molecules (34). Estrogen deficiency can change the expression of estrogen target genes and increase the secretion of bone metabolic factors such as IL-1, IL-6, and TNF-α (16). The over-expression of inflammation and bone rarefaction were detected in OVX mice also with higher TNF-α level (35, 36). TNF receptor (TNFR)1 and TNFR2 released by adipose tissue were activated by TNF-α. Apoptosis or survival signaling may be induced by TNFRs signaling. TNFRs Signaling in osteoclasts is generally proliferative, whereas signaling in osteoblasts is inhibitory (37). A study by Wang suggested that TNF-α acts at multiple sites in the RANK–RANKL pathway (38). The variety of the femoral neck BMD is correlated significantly with that of soluble (s)TNFR1 (33). The reason for this analysis is that estrogen deficiency in OVX rats increased bone resorption, while exercise reduced adipose tissue, activated TNF-α-mediated signaling pathway, and increased bone formation, thereby improving bone mineral density in OVX + EX rats.

In recent years, studies have demonstrated that the epigenetic mechanism is related to osteogenic differentiation, osteogenesis, bone remodeling, and other processes related to bone metabolism (39, 40). Abnormal epigenetic regulation can lead to a series of diseases related to bone metabolism, such as osteoporosis. The present study revealed that the protein expressions of HDAC8, LRTOMT, TGS1 and ANKRD46 in bone tissue were closely related to bone microstructure. The protein expressions of HDAC 8, LRTOMT and TGS1 in the femur tissue of OVX rats were significantly downregulated, while ANKED46 was significantly upregulated after 17 weeks of moderate-intensity treadmill exercise. Kim et al. found that interval treadmill training can improve muscle growth and osteogenic differentiation by enhancing the expression of bone morphogenetic proteins and deacetylase in OVX rats (41). Other studies have shown that HDACs regulate bone development and inhibits the osteogenic differentiation of bone marrow stromal cells by removing the acetylation of histone H3 lysine 9 leading to transcriptional inhibition (42–44). Similarly, evidence suggests that electroacupuncture improves the BMD and trabecular morphology of OVX-induced osteoporosis by downregulating the expression of HDAC2 and promoting the acetylation of histone H3 (45). Only a few studies have been conducted on LRTOMT, but homology has been identified between LRTOMT and catechol O-methyltransferase (COMT), with COMT being involved in estrogen inactivation. One study identified that COMT gene polymorphism is involved in the regulation of the association between physical activity and BMD (46). Shao et al. reported that green tea polyphenols could protect against bone loss in OVX rats by reducing the expression of COMT (47). The present study suggested that treadmill exercise might regulate the expression of LRTOMT through acetylation modification and methylation modification, HDAC 8 and LRTOMT might be involved in the metabolism of estrogen in bone tissues, thus affecting bone metabolism.

TGS1 mediates the formation of trimethylguanosine caps on a variety of Rnas, including snRNAs and telomerase Rnas. In vitro experiments showed that TGS1 inhibited telomerase levels and limited telomere elongation (48). ANKED46 encodes Ankyrin Repeat domain 46, involved in the regulation of protein, cell proliferation, cell apoptosis, cell adhesion and migration. Increased expression of ANKED46 inhibits cell proliferation, migration and tumor growth (49). There is no report on Tgs1 and ANKED46 in bone. In this study, we found that TGS1 and ANKED46 were closely related to bone remodeling. Exercise decreased TGS1expression and increased ANKED46 expression in femur of OVX rats. The mechanism of their involvement in bone remodeling remains to be studied.

At present, a variety of omics technologies are being applied to the mechanism underlying PMOP (50). But no single technique is able to fully identify the pathological molecular mechanisms involved. With the advancement in omics technologies, the integration of multi-omics data should be able to provide a clearer and more complete view of the role of exercise in delaying the pathogenesis of PMOP.

In conclusion, moderate-intensity treadmill exercise improves the body composition, bone mass, and bone metabolism of OVX rats through the TNF-α-mediated signaling pathway. The differentially expressed proteins in the femur, such as HDAC8, LRTOMT, TGS1 and ANKRD46 may be important targets for moderate-intensity treadmill exercise to improve bone mass and aid in the prevention and treatment of PMOP.

## Data Availability

The original contributions presented in the study are included in the article/Supplementary Material, further inquiries can be directed to the corresponding author/s.
